# Virtual Reality Breathing Interventions for Mental Health: A Systematic Review and Meta-Analysis of Randomized Controlled Trials

**DOI:** 10.1007/s10484-023-09611-4

**Published:** 2024-01-18

**Authors:** Gabriela Cortez-Vázquez, Marcel Adriaanse, George Louis Burchell, Raymond Ostelo, Georgia Panayiotou, Elke Vlemincx

**Affiliations:** 1 Department of Health Sciences, Vrije Universiteit Amsterdam, Amsterdam, The Netherlands; 2 Medical Library, Vrije Universiteit Amsterdam, Amsterdam, The Netherlands; 3 Department of Psychology and Center for Applied Neuroscience, University of Cyprus, Nicosia, Cyprus; 4 Amsterdam Public Health Research Institute, Amsterdam, The Netherlands; 5 Amsterdam Movement Sciences Research Institute, Amsterdam, The Netherlands; 6 Department of Epidemiology and Data Science, Amsterdam UMC location, Vrije Universiteit, Amsterdam, The Netherlands; 7 Health Psychology, KU Leuven, Leuven, Belgium

**Keywords:** Virtual reality, Breathing interventions, Mental health, Systematic review, Meta-analysis

## Abstract

Breathing exercises have been shown to reduce mental health problems among clinical and non-clinical populations. Although virtual reality (VR) breathing interventions are assumed to have potential benefits, it remains unclear whether VR breathing interventions are more effective at improving mental health than non-VR breathing interventions. We conducted a systematic literature search in six electronic databases (Web of Science, PsycINFO, Embase, Cochrane Central Register of Controlled Trials, Scopus, and PubMed) from inception to 30th September, 2022. We included randomized controlled trials in adults evaluating effects of VR compared to non-VR breathing interventions on primary outcomes of mental health (stress, anxiety and mood), and secondary outcomes of physiological stress measures (e.g., heart rate (HR), heart rate variability (HRV)). Within these selected studies, we explored differences in likeability and future use between VR and non-VR breathing interventions. 2.848 records were identified of which 65 full-text articles were assessed. Six RCTs were included, of which five were suitable for meta-analyses. Comparing VR to non-VR breathing interventions, there were no significant differences in overall mental health, stress, anxiety or mood, nor in HR or HRV. There was no evidence that participants liked VR breathing interventions more than non-VR, nor would use them more in the future. These results suggest that there is no evidence that VR breathing interventions are more effective than non-VR in improving mental health outcomes, HR, HRV. Further research is required to determine whether there may be advantages to longer-term VR-implementation and practice, and explore possible mechanisms.

**Supplementary Information:**

The online version contains supplementary material available at 10.1007/s10484-023-09611-4.

## Introduction

Breathing interventions have gained popularity as evidence-based strategies for preventing both physical and mental illness, and promoting mental health (Fried & Grimaldi, 1993). Breathing interventions can generally be divided into two categories: breath awareness and breath control. Breath awareness (e.g., mindful breathing) refers to paying attention to, and raising awareness of, the breath without attempting to control it (Arch & Craske, 2006; Ospina et al., 2007; Pozuelos et al., 2019). Breath control (or breath work) involves consciously altering the frequency of the breath, depth of the breath, the inhalation/exhalation ratio (I/E ratio), and/or the location of breath (movement of the abdomen vs. chest, or nose vs. mouth) (Fincham et al., 2023; Zaccaro et al., 2018). Different techniques exist to control breathing. Paced slow breathing aims to reduce breath rate by voluntary matching breathing to a set breath rate less than ten breaths per minute (Zaccaro et al., 2018), but often approximately six breaths per minute (Laborde et al., 2022), using a pacer. Diaphragmatic breathing involves breathing deeply and slowly from the diaphragm (Hopper et al., 2019) with or without pacer. Respiratory biofeedback uses real-time visualizations of objectively measured respiratory parameters, such as breathing frequency or diaphragm activity, to train the modification of respiration. More popular than respiratory biofeedback, is heart rate variability (HRV) biofeedback in which participants aim to maximize heart rate variability based on animated feedback of real-time heart rate variability, most often by breathing slowly (Yu et al., 2018; for reviews see De Witte et al., 2019; Goessl et al., 2017; Lehrer et al., 2020; Pizzoli et al., 2021). Real-time feedback of respiration and HRV is typically provided through two-dimensional (2D) screen-based displays.

The most studied breathing intervention for physical and mental health is slow breathing (Fincham et al., 2023). By stimulating vagally mediated activity of the parasympathetic nervous system (Gerritsen & Band, 2018; Lehrer et al., 2020), slow breathing interventions may improve physical and mental health, and alleviate physical and mental health complaints associated with sympathetic nervous system dominance (Jerath et al., 2015). For example, slow breathing compared to active controls such as neurofeedback, sham breathing, and exercise rehabilitation, as well as inactive control groups including breathing naturally, treatment-as-usual (TAU), reading a magazine or no intervention have reduced blood pressure and heart rate (HR), and increased HRV among cardiovascular disease patients (Lehrer et al., 2020; Telles et al., 2013; Yau & Loke, 2021; Zou et al., 2017) and healthy adults (Gholamrezaei et al., 2021; Lehrer et al., 2020). It has also improved mood compared to active controls such as mindfulness and ludic cognitive activities (Balban et al., 2023; Novaes et al., 2020), and reduced symptoms in panic disorder and post-traumatic stress disorder compared with TAU or other active controls, including exposure, progressive muscle relaxation, and sham HRV-Biofeedback (Banushi et al., 2023; Blase et al., 2021). Additionally, it has reduced physiological and psychological stress (Brown et al., 2013; Fincham et al., 2023; Goessl et al., 2017; Laborde et al., 2022), and depression and anxiety (Blase et al., 2021; Goessl et al., 2017; Hopper et al., 2019; Lehrer et al., 2020; Yu et al., 2018) compared with both active and inactive controls, including cognitive tasks, psychoeducation, meditation, relaxation, sham biofeedback, standard care, waiting-list, and within-group designs. Furthermore, it has increased protective factors of mental health, such as relaxation and positive mood (Van Diest et al., 2014), interoceptive awareness (Leganes-Fonteneau et al., 2021), and flexibility (Van Diest et al., 2014) in within-group designs.

Virtual reality (VR) technology with head-mounted displays has recently emerged as an innovative method of implementing breathing interventions (breath awareness and breath control), because of its potential advantages over non-VR implementation. First, VR provides a fully immersive experience that may facilitate engagement and help increase long-term adherence (Rockstroh et al., 2019). Additionally, participants are more likely to actively engage with the immersive virtual environment (e.g., via game features), enhancing their motivation to practice (Al-Rayes et al., 2022) and consequently increasing their mastery of breathing techniques (Blum et al., 2020). Second, VR implementation can mitigate the challenges associated with breathing interventions. In many cases, participants have difficulty following breathing intervention instructions (e.g., finding the right breathing volume and/or I/E ratio) or maintaining attention to the breathing. As a result of incorrectly performing breathing control, adverse effects can occur, including hyperventilation or hyperactivation of the parasympathetic system (Jerath et al., 2006). Instead, VR-based breathing interventions can reduce the complexity of breathing techniques through the incorporation of gamification. Gamified feedback may involve a greater sense of control and self-efficacy (Cheng & Ebrahimi, 2023; Weerdmeester et al., 2020), supporting the execution of breathing techniques (Shih et al., 2020). Third, the immersive experience may minimize external distractions such as environmental noise and visual interruptions, as well as internal distractions such as interpretations or judgments of bodily sensations, mind wandering or disruptive cognitions. As a result, increased attentional focus on the breathing exercises (Lüddecke & Felnhofer, 2022) and a heightened sense of presence within the virtual environment (Cummings & Bailenson, 2016) can help to maximize skills training (Hamilton et al., 2021).

In line with these potential advantages, evidence has suggested that VR breathing interventions may increase relaxation in pre-post designs (Fominykh et al., 2018; Kosunen et al., 2016; Rockstroh et al., 2021) and self-efficacy, both in pre-post designs and in comparisons with non-VR breathing interventions (Rockstroh et al., 2021; van Rooij et al., 2016; Weerdmeester et al., 2021). Additionally, they may reduce anxiety when compared to non-VR breathing interventions, TAU, or in single-case experiments (Bossenbroek et al., 2020; Prabhu et al., 2020; Venuturupalli et al., 2019; Weerdmeester et al., 2021). Furthermore, they may reduce stress (Cook et al., 2021; Rockstroh et al., 2021) and negative mood both in pre-post designs, and when compared to meditation or watching a nature video (Cook et al., 2021; Naylor et al., 2019). Accordingly, they have also resulted in lowered respiration rate (Prabhu et al., 2020) and increased HRV (Aganov et al., 2022; Prabhu et al., 2020) when compared with TAU and sham VR conditions. However, some studies have suggested that VR-based interventions are equally effective as non-VR breathing interventions for mental health (Blum et al., 2019; Rockstroh et al., 2019; Tinga et al., 2019; Weerdmeester et al., 2021).

Furthermore, VR breathing interventions have also yielded a satisfying post-intervention user experience, with participants finding them highly engaging and likeable (Blum et al., 2020; Cook et al., 2021; van Rooij et al., 2016). Additionally, such interventions have proven to be more interesting and enjoyable when compared to a 2D nature video condition (Naylor et al., 2019). Nevertheless, some studies have reported poor user experiences, as participants may become more distracted and less engaged, particularly with specific elements of the VR breathing intervention such as biofeedback (Hendriks & Rombout, 2018). Participants may also experience negative effects such as claustrophobia and boredom (Naylor et al., 2019), and mild dizziness and nausea (Cook et al., 2021).

Previous research seems to suggest that the benefits of VR breathing interventions are superior to those of non-VR. However, previous meta-analyses have focused on different VR-based mental health interventions with no emphasis on breathing interventions. For example, a meta-review showed that VR-based mental health interventions such as biofeedback, exposure therapy and cognitive behavioral therapy, improve depression, anxiety, stress-related and psychiatric disorders compared with inactive control groups (waiting-list, placebo [e.g., attentional control], and TAU). Nonetheless, the effects of these are similar to those of non-VR standard treatments such as evidence-based therapy, relaxation techniques, and exposure therapy (Dellazizzo et al., 2020). Interestingly, VR-based interventions may show more enduring effects in the long-term follow-up (> 3 months) than active controls for panic disorder and aviophobia (Dellazizzo et al., 2020). Furthermore, previous studies have tested the effectiveness of different VR-based stress management interventions which, however, did not allow to isolate the effects of VR-breathing interventions. For example, a meta-analysis (which included randomized controlled trials [RCTs] and non-randomized designs) found that different VR-based biofeedback interventions including meditation, HRV-biofeedback, breath awareness, and relaxation techniques (muscle relaxation and autogenic training) significantly reduced anxiety and HR, yet did not significantly increase HRV. Nevertheless, results from RCTs indicated no significant differences in anxiety, HR, and HRV between VR and non-VR 2D biofeedback, and no significant differences between VR and waiting-list in anxiety and HRV (Kothgassner et al., 2022). Therefore, evidence on the effectiveness of VR breathing interventions on mental health is inconclusive. Yet, some studies suggest the potential advantages of VR regarding user experience, likeability and motivation (Velana et al., 2022).

The present study systematically reviews and quantifies the effectiveness of VR breathing interventions in adults, as compared with non-VR breathing interventions, on primary mental health outcomes of stress, anxiety and mood, and secondary outcomes of physiological measures of stress, likeability and future use. The study pools findings from randomized controlled studies only. This way, we intend to gain a better understanding of the superior benefits of VR breathing interventions over non-VR breathing interventions for mental health.

## Methods

### Protocol and Registration

**Fig. 1:**
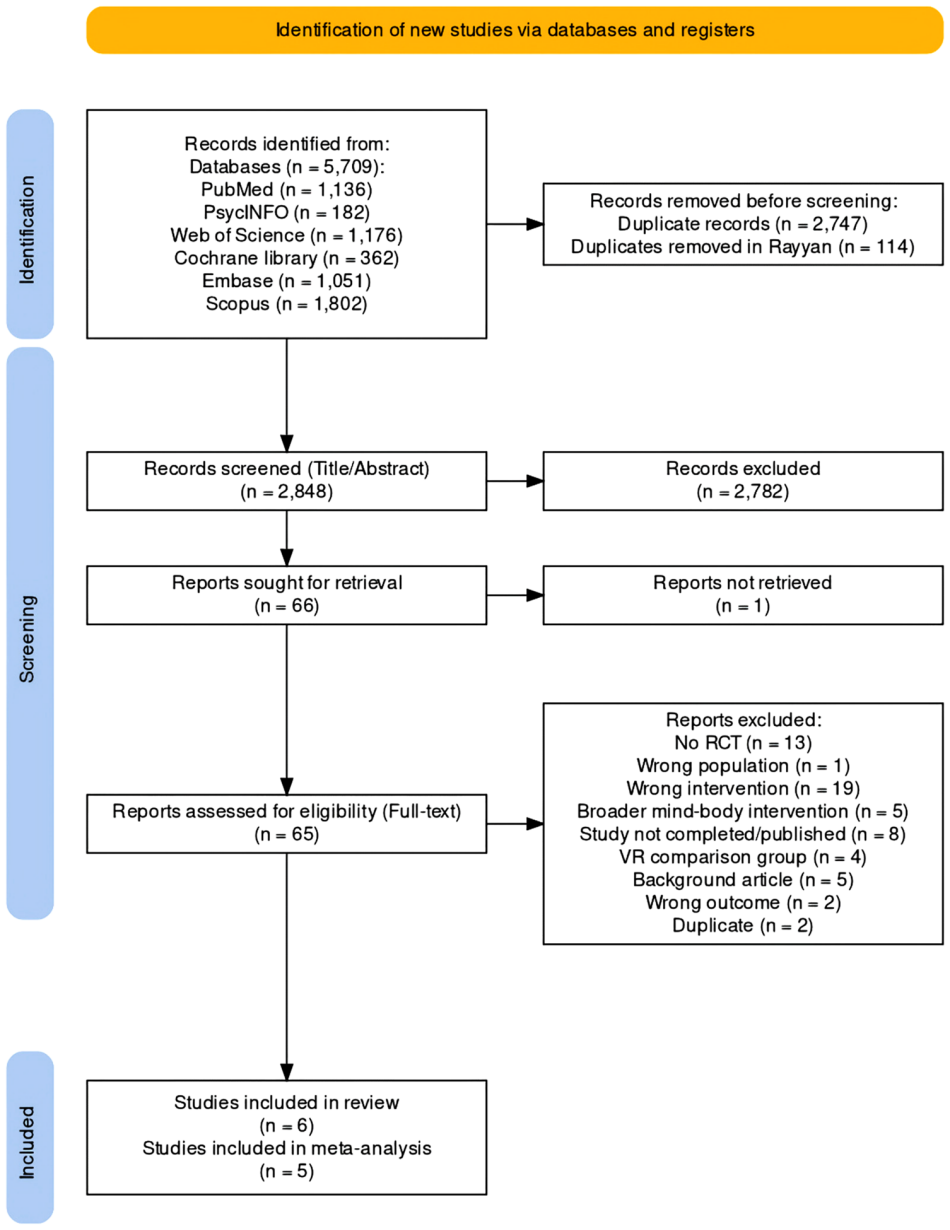
PRISMA 2020 flow diagram of study selection. *Note*: This figure was created and adapted using the online PRISMA Flow Diagram Tool (Haddaway et al., 2022).

This study analyzed experimental studies evaluating the effects of VR breathing interventions over non-VR breathing interventions for mental health outcomes. This systematic review and meta-analysis is reported following the Preferred Reporting Items for Systematic Reviews and Meta-analyses (PRISMA) guidelines and used a relevant checklist for the development of the study protocol, the conduct of the study, and drafting the manuscript (Page et al., 2021) as seen in Fig. 1. We registered the study protocol on the PROSPERO database; registration number CRD42021265506.

### Search Strategy

A systematic search was performed in the databases: PubMed, PsycINFO, Clarivate Analytics/Web of Science Core Collection, Cochrane library, Embase and Scopus. The timeframe within the databases was from inception to 30th of September 2022 and conducted by GLB, GCV and EV. The search included 10 keywords and free text terms^1^ for (synonyms of) ‘virtual reality’ combined with (synonyms of) ‘breathing exercises’. Since indexed terms^2^, such as Medical Subject Headings (MeSH) employed in PubMed, are not standardized across the databases, we used equivalent indexed terms to ensure consistency. A full overview of the search terms per database can be found in the supplementary information (see supplementary Tables S2-S7). No limitations on date or language were applied in the search.

Duplicate articles were excluded using the R-package “ASYSD” (an automated deduplication tool; Hair et al., 2021) followed by manual deduplication in Endnote (X20.0.3) by the medical information specialist (GLB). To identify any additional relevant studies, we systematically screened reference lists of key systematic reviews that were retrieved from the search string that was originally used as an orientation on currently available review topics.

### Inclusion and Exclusion Criteria

We included RCTs published in English. Uncontrolled trials, and non-randomized trials were excluded. There were no restrictions on the publication period. Unpublished studies were not specifically searched. We included dissertations and conference abstracts.

#### Participants

We included healthy, sub-clinical and clinical populations of adults aged ≥ 16 to 60 years. This age range was selected to minimize the potential influence of age-related changes on physiological and mental health outcomes, including alterations in the autonomic nervous system activity (Billman et al., 2019; Voss et al., 2015). Furthermore, we considered the added complexity associated with the use of VR breathing interventions and the specific challenges encountered in children and the elderly, such as heightened dizziness, fatigue, disorientation, and nausea (Kaimara et al., 2022; Liu et al., 2020; Plechatá et al., 2019).

#### Intervention

We focused on isolated breathing interventions delivered via immersive VR. Possible breathing interventions included: (1) breath awareness/attention (e.g., mindful breathing, focused attention to the breathing, count breathing); and/or (2) breath control including paced breathing, respiratory biofeedback, diaphragmatic breathing, pranayama breathing, HRV biofeedback, resonant frequency breathing, (alternate) nostril breathing, and Buteyko breathing.

We excluded breathing interventions that were embedded in a broader intervention targeting not mainly breathing but primarily other mind-body methods or psychological treatments, in order to ensure the isolated effects of breathing interventions. This approach was taken to avoid potential confounding of the effects of breathing interventions when combined with methods that include other working mechanisms, including posture and balance (e.g., Asanas yoga, Qigong, Pilates, Tai Chi), energy (e.g., Kundalini meditation), analytical meditation (e.g., Vipassana, compassion, loving-kindness meditation), spiritual growth and/or altered consciousness or awareness of object aside from breathing (e.g., transcendental meditation, mantra meditation, chanting), meditation techniques other than only breath meditation (e.g., visualization, mindfulness), progressive muscle relaxation and/or neurofeedback, and psychological treatment (e.g., cognitive behavior therapy, acceptance and commitment therapy). Furthermore, we excluded interventions targeting respiratory muscle training, as they mainly target respiratory functions (e.g., in respiratory or neurodegenerative disorders) rather than mental health.

#### Control Condition

Eligible studies included an inactive or active control group that did not receive any alternative VR exposure (e.g., non-VR implementation of breathing interventions such as two-dimensional screen implementation, waiting-list, no treatment, and/or placebo). We excluded studies that did not have any control group or used a different VR exposure as a control condition.

#### Primary Outcomes

Primary mental health outcomes include psychological self-reports, clinical, and/or behavioral measurements that assess general distress, stress, anxiety, depression, mood, and general mental and physical health-related symptoms (e.g., general health questionnaire, health status questionnaire). Mental health has been defined as an overall state of wellbeing that enables individuals to effectively cope with the challenges of daily life, function independently, and contribute positively to their communities (World Health Organization, 2004). This study focused exclusively on mental health outcomes related to affect and stress-related responses including stress, depression, anxiety, distress and mood. These outcomes were chosen because of the psychophysiological mechanisms underlying associations between breathing and these outcomes (as discussed in the introduction). We focused on mental health as an integrated construct comprising of stress, depression, anxiety, distress and mood, and on these different outcomes independently for the following reasons. Prior research has shown that stress can contribute to depression and anxiety (Godoy et al., 2018; Tafet & Nemeroff, 2016). Anxiety and depression have distinctive characteristics, yet they are often comorbid (McGrath et al., 2020; Saha et al., 2021; ter Meulen et al., 2021), and both can affect emotional states or mood (Norton et al., 2005; Norton & Mehta, 2007; Paulus et al., 2015; Toro Tobar et al., 2020), thus sharing a number of similar symptoms. Therefore, we aim to evaluate the *overall* effect on mental health outcomes combined, while also examining differences in each mental health outcome separately.

#### Secondary Outcomes

We included neurobiological and physiological measures of stress, including autonomic stress responses (e.g., HR, HRV, respiratory sinus arrhythmia, galvanic skin response, electromyography) and hypothalamus-pituitary-adrenal axis outcomes (e.g., cortisol), as well as protective factors for mental illness, such as self-efficacy, flexibility, resilience, emotion regulation and coping skills, as secondary outcomes.

### Data Collection and Analysis

#### Study Selection

We screened articles for eligibility using the Rayyan screening tool (Ouzzani et al., 2016). First, GCV and two reviewers (JG and TZG) independently screened articles based on title and abstract. Next, potentially eligible articles were independently reviewed in full-text by GCV, and two reviewers JG and TZG for a definite inclusion. Decisions were blinded. Disagreements were resolved by a third reviewer (EV). Reviewers verified and eliminated duplicate entries when the Rayyan tool identified them.

#### Data Collection

We designed an excel spreadsheet template based on the Cochrane “Data collection form for intervention reviews: RCTs and non-RCTs” (Higgins et al., 2022). Data extraction was performed independently by two authors (GCV and EV). Discrepancies were discussed and solved by GCV and EV. The extracted data included: publication characteristics (author, year, country); participants characteristics (gender, age, total of participants enrolled in the study and subgroups); intervention (intervention components/description and setting, number of sessions, duration of sessions, breathing exercises); comparator(s) characteristics (control components/characteristics and setting, type of comparator, breathing exercises); outcomes measures (measurement tool, effects tested, type of analyses, main findings). For each group, we extracted *n*, mean scores and *SD* at different time points available (pre, during, post, follow-up). When it was not possible to extract the required data, we contacted the (corresponding) authors. When data could not be obtained at post, we excluded the articles (n = 1) from the quantitative synthesis.

#### Data Extraction

For the primary outcomes, we extracted continuous outcomes of mental health, including self-reported trait and state anxiety, positive and negative mood, stress and relaxation. For the secondary outcomes, we retrieved outcomes of self-reported self-efficacy and relaxation self-efficacy, and physiological measures of stress: HR and HRV (standard deviation of normal-to-normal intervals [SDNN], root mean square of successive differences [RMSSD], coherence ratio, low-frequency to high-frequency ratio, low frequency). We did not retrieve cognitive-related outcomes such as attention to the present moment, mindfulness (non-judgmental awareness), mind wandering, or flow as our main focus was outcomes directly related to affect. Although we initially did not consider evaluation and experience-related outcomes (e.g., user experience, intent to use, liking), we chose to include them in order to explore differences between VR breathing interventions and comparators in the selected studies. Thus, we retrieved any continuous outcome that measured how participants felt about the intervention, such as enjoyment, liking, intention to use and recommendation. When studies reported more than one measure of the same outcome (e.g., sub-scale scores, different measures of the same outcome, as well as measures for both active and control groups), we coded them as individual entries within each study and accounted for the dependency of the data with a multi-level model analysis.

#### Risk of Bias Assessment

The Cochrane Risk of Bias tool 2.0 (RoB) was used to assess the methodological limitations of the included randomized trials (Sterne et al., 2019). RoB assesses bias arising from five domains: (1) randomization process, (2) deviation of from intended interventions, (3) missing outcome data, (4) measurement of the outcome, and (5) selection of the reported results. GCV completed an introductory overview of RoB accessible at https://training-cochrane-org.vu-nl.idm.oclc.org/resource/introducing-rob-2. Additionally, EV and GCV independently completed the assessment of RoB for each included study by referring to the full guidance document available at https://sites.google.com/site/riskofbiastool/. Initial disagreements were discussed among GCV and EV. If a consensus could not be reached, RO was consulted to reach agreement.

#### Quality Assessment

The overall quality and uncertainty of the evidence were assessed using the Grading of Recommendations Assessment, Development, and Evaluation (GRADE) (Guyatt et al., 2008) by three reviewers (GCV, EV, RO). GCV and EV initially rated the importance of each pooled outcome and assessed them based on five GRADE domains: (1) risk of bias, (2) imprecision, (3) indirectness, (4) heterogeneity, and (5) publication bias. Possible ratings for each pooled outcome were either high, moderate, low or very low, representing the strength of the evidence (Guyatt et al., 2008). GCV discussed initial ratings with RO. Consequently, through discussion, GCV and EV reached a consensus on the overall GRADE ratings.

#### Data Synthesis

As our main interest was to evaluate the effects of breathing interventions on mental health outcomes compared with non-VR implementation, we only included comparisons between VR to active control groups in the meta-analysis. Therefore, we excluded extracted effect sizes based on inactive control groups (n = 1)^3^. We calculated the standardized mean difference (SMD) assuming unequal variances between the groups (Bonett, 2009) using *mean scores*, *SD*s, and *n.* To ensure consistency across studies, we calculated SMDs at post-intervention for mental health and experience-related outcomes, and during breathing interventions for physiological outcomes. This allowed us to compare immediate intervention effects while avoiding the influence of extraneous factors (e.g., additional interventions, stress inductions, recovery times). Weerdmeester et al. (2021) exposed participants to two distinct training phases: exposure to relaxing and stressful VR environments. For each training phase, separate effect sizes were calculated to quantify the effect of each exposure. Since Hu et al. (2021), exposed participants to both VR mindful breathing and home-based diaphragmatic breathing, effect sizes were computed only after *the first* VR mindful breathing exposure (day 1) in order to isolate VR mindful breathing effects^4^. Since only one study reported data at follow-up (> 1 month), pooling data was not possible. Effect sizes were quantified such that a positive effect size favored the intervention group, and a negative effect size favored the control group. Effect sizes are numbered in all tables and figures by effect size identification (ES ID). SMDs of 0.2, 0.5, and 0.8 are considered small, medium, and large effects, respectively (Cohen, 1988).

#### Statistical Analyses

We used a multi-level meta-analysis approach to account for the dependence among multiple effect sizes within studies (Cheung, 2019; López-López et al., 2018; Van Den Noortgate & Onghena, 2003). Therefore, we avoided losing relevant data when aggregating multiple effect sizes or selecting one effect size per study (Cheung, 2019). Three levels of variance were considered in our meta-analysis: sampling variance (level 1), variation between effect sizes of the same study (level 2: within-study variance, or between-outcomes variance), variance in effect sizes between studies (level 3: between-study variance) (Fernández-Castilla et al., 2020). Restricted maximum-likelihood (REML) method was used to estimate the model parameters. 95% confidence intervals (CI) and the test of individual coefficients were based on a *t*-distribution (Viechtbauer, 2010).

For *overall* mental health, we pooled effects sizes of mood, stress and anxiety. We also inspected each outcome separately in sub-group analyses when possible. In addition, we ran a sub-group analysis for HRV-biofeedback interventions. For secondary outcomes, we pooled available effect sizes of physiological outcomes into (1) HR, (2) RMSSD, and (3) SDNN^5^ respectively. Experienced-related outcomes were pooled into (1) liking and (2) future use. Statistical heterogeneity between studies was assessed through forest plots, the Q-statistic, tau-squared (*τ*^*2*^), and I^2^. *τ*^*2*^*and* I^2^ are quantified for each level of variance as defined above. Subgroup analyses were only performed if subgroups consisted of two or more effect sizes. Finally, we examined whether individual effect sizes were outliers and/or influential in the models with studentized residuals and Cook’s distances (Viechtbauer & Cheung, 2010). A funnel plot was inspected and Eggers’s test of the intercept including standard error as moderator was used to assess whether there was a risk of publication bias. For all analyses, we used R (version 4.2.1) (R Core Team, 2022), RStudio (version 2022.12.0 + 353) (RStudio Team, 2022) and the metafor package (version 3.4.0) (Viechtbauer, 2010).

## Results

### Search and Study Selection

Our search resulted in 5,709 articles. Following the removal of 2,861 duplicate records, we retrieved 2,848 papers for further screening. We excluded 2,782 records based on title and abstract, and retained 65 records for full-text article screening. Finally, we included six studies meeting the inclusion criteria in the qualitative synthesis (Blum et al., 2019; Hu et al., 2021; Rockstroh et al., 2021; Waller et al., 2021; Weerdmeester et al., 2021; Weibel et al., 2023). The cross-over trial (Waller et al., 2021) was excluded from the meta-analysis since it only reported paired analyses of both periods^6^. Therefore, five studies were included in the meta-analyses (see Fig. 1).

### Study Characteristics

We included five parallel-group RCTs (Blum et al., 2019; Hu et al., 2021; Rockstroh et al., 2019; Weerdmeester et al., 2021; Weibel et al., 2023) and one RCT cross-over (Waller et al., 2021) that examined the effects of VR-based breathing interventions on mental health outcomes. These RCTs included a total of 469 participants between 18 and 43 years old (mean = 25.52, *SD =* 4.63, 62.2% females). 76.1% (n = 357) were healthy participants who work or study, and 23.9% (n = 112) were undergraduate students with high levels of stress and anxiety.

Five studies compared a VR breathing intervention with a non-VR breathing intervention (active control group). Only one study included both inactive and active control groups. Three out of six studies compared the impact between one session of VR HRV-biofeedback with slow breathing without pacer and a non-VR HRV-biofeedback using 2D abstract graphics (Blum et al., 2019; Rockstroh et al., 2019) or nature-inspired backgrounds (Weibel et al., 2023). Additionally, Weibel et al. (2023) compared a single session of VR-based slow paced breathing to a non-VR 2D screen display paced breathing. Another study compared four sessions of VR game-based diaphragmatic breathing with and without stress exposure to non-VR paced breathing via a smartphone app (Weerdmeester et al., 2021), including a three-month follow-up. Hu et al. (2021) compared VR mindful breathing (attention to the sensations of breathing) to non-VR traditional mindful breathing, both before and after 5-day home training of diaphragmatic slow breathing. Finally, in a cross-over design, VR mindful breathing was compared with non-VR mindful breathing delivered through either face-to-face interaction or a 2D pre-recorded video screen (Waller et al., 2021) (see Table 1).

**Table 1:** Study characteristics

Author (year) [Country]	Design	Population	N	M_age_ (SD) [% female]	Intervention	Breathing exercises	Number of sessions (Duration of breathing exercise)	Time point [Follow-up]	Control group [Type]	Effect Size ID	Outcomes	Scale
Blum et al. (2019) [Germany]	RCT parallel groups	Healthy participants	60	33.5 (9.4) [51.7%]	VR HRV-biofeedback	Paced slow breathing at 6 br/min with a 5:5 I/E ratio	1 session (10 min)	Post [N/A]	Non-VR 2D HRV biofeedback with paced slow breathing at 6 br/min with a 5:5 i/e ratio [Active]	1	**Relaxation**	STAI
										2	**NA**	VAS
										3	**HR**	
										4	**RMSSD**	
										5	**Liking**	VAS
Hu et al. (2021) [USA]	RCT parallel groups	Healthy participants	40	28 (4) [52%]	VR mindful breathing + diaphragmatic slow breathing at home	Breathing awareness + diaphragmatic slow breathing at 6 br/min with 3:6:1 I/E ratio	7 sessions: 2 lab sessions (10 min) 5 home sessions (5 min, 3x per day)	Post [N/A]	Traditional mindful breathing + diaphragmatic slow breathing at home [Active]	1	**PA**	PANAS
										2	**NA**	PANAS
										3	**SDNN**	
Rockstroh et al. (2019) [Germany]	RCT parallel groups	Healthy participants	68	22.9 (4) [60.3%]	VR HRV-biofeedback	Paced slow breathing at 6 br/min	1 session (10 min)	Post [N/A]	Non-VR 2D HRV biofeedback with paced slow breathing at 6 br/min [Active]	1	**Good mood**	GMMQ
										2	**Alertness/wakefulness**	GMMQ
										3	**Rest/calm**	GMMQ
										4	**HR**	
										5	**RMSSD**	
										6	**SDNN**	
										7	**Liking**	VAS
										8	**Future use**	VAS
										9	**Recommend**	VAS
								Post [N/A]	Neutral nature movie with instructions to relax [Inactive]		Good mood	GMMQ
											Alertness/wakefulness	GMMQ
											Rest/calm	GMMQ
											HR	
											RMSSD	
											SDNN	
											Liking	VAS
Waller et al. (2021) [Canada]	RCT cross-over	Healthy participants	82	17 to 28 (range) [61%]	VR mindful breathing	Breath awareness	1 session (5 min)	Post [N/A]	Non-VR face-to face mindful breathing [Active]		PA	mDES
											NA	mDES
											Affect states	BASS
											Mood experiences	MEQ
											Satisfaction	SCQ
								Post [N/A]	Non-VR 2D pre-recorded mindful breathing [Active]		PA	mDES
											NA	mDES
											Affect states	BASS
											Mood experiences	MEQ
											Satisfaction	SCQ
Weerdmeester et al. (2021) [Netherlands]	RCT parallel groups	Under-graduate students with elevated anxiety and/or stress	112	20.71 (2.46) [90.2%]	VR game-based biofeedback of diaphragmatic breathing	Diaphragmatic breathing (not paced)	2 training phases of 2 sessions each (10 min)	Post [3-month]	Diaphragmatic paced breathing App at 6 br/min with 3:7 i/e ratio [Active]	1,2	**State Anxiety**	STAI
										3	**Enjoyment**	10-point scale
										4	**Future use**	4-point scale
										5	**Recommend**	4-point scale
Weibel et al. (2023) [Switzerland]	RCT parallel groups	Healthy participants	107	22.52 (3.33) [41%]	VR HRV biofeedback	HRV with slow paced breathing at individual resonance frequency (4.5–6.5 br/min)	1 session 2 blocks (10 min)	Post [N/A]	non-VR 2D HRV biofeedback [Active]	1	**Good mood**	MMQ
										2	**Alertness/wakefulness**	MMQ
										3	**Rest/calm**	MMQ
										4	**Relaxation**	VAS
										5	**Stress**	VAS
										6	**HR**	
										7	**SDNN**	
										8	**Future use**	UTAUT
										9	**Liking**	UTAUT
					VR Standardized paced breathing	Paced breathing at 6 br/min	1 session 2 blocks (10 min)	Post [N/A]	Non-VR 2D standardized paced breathing [Active]	10	**Good mood**	MMQ
										11	**Alertness/wakefulness**	MMQ
										12	**Rest/calm**	MMQ
										13	**Relaxation**	VAS
										14	**Stress**	VAS
										15	**HR**	
										16	**SDNN**	
										17	**Future use**	UTAUT
										18	**Liking**	UTAUT

*Note*: N = total sample; Mage = mean age; SD = standard deviation; VR HRV-biofeedback = virtual reality heart rate variability biofeedback; VR = virtual reality via head mounted display; br/min = breath per minute; I/E ratio = inhalation/exhalation ratio; Non-VR 2D = 2D standard display; Relaxation = subjective relaxation; NA = negative affect; PA = Positive affect; HR = heart rate; RMSSD = root mean square of the successive differences; SDNN = standard deviation of normal to normal RR intervals; STAI = state anxiety inventory; VAS = visual analogue scale; PANAS = positive and negative affect schedule; GMMQ = German multidimensional mood questionnaire; mDES = modified differential emotions scale; BASS = Buddhist affective states scale; MEQ = meditative experiences questionnaire; SCQ = satisfaction and credibility questionnaire; MMQ = multidimensional mood questionnaire; UTAUT = unified theory of acceptance and use of technology questionnaire. The outcomes used in the meta-analyses are indicated in bold

### Risk of Bias Assessment

**Fig. 2:**
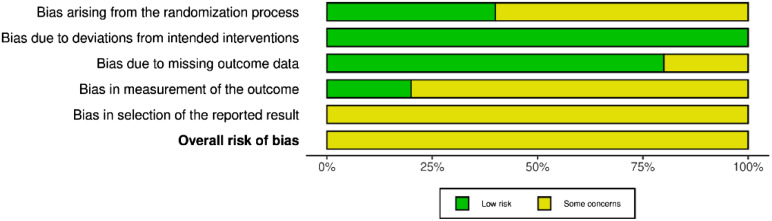
Summary figure showing the proportion of studies with specific levels of risk of bias in each domain according to the Cochrane Risk of Bias Assessment 2.0. *Note*: This figure was created and adapted using the online Risk-of-bias Visualization (robvis) tool (McGuinness & Higgins, 2020).

All five parallel-group RCTs raised some concerns of bias (see Fig. 2)^7^. The randomization procedure and allocation concealment in three studies (Hu et al., 2021; Weerdmeester et al., 2021; Weibel et al., 2023) raised some concerns regarding bias. There were, however, no baseline differences across conditions. One study blinded both participants and experimenters (Blum et al., 2019), and one study blinded only experimenters (Rockstroh et al., 2019). The nature of the interventions prevented blinding of participants and experimenters in three studies. However, there was no deviation from the intended interventions and impact on the outcomes, resulting in a low risk of bias. All studies used intention-to-treat analyses, however, one study raised concerns due to the high percentage of missing data (Weerdmeester et al., 2021). Due to the lack of blinding of participants in four studies, there was a risk of bias in self-reported mental health outcomes. The physiological measures demonstrated a low risk of bias. In all studies, there was no information on prespecified data analyses, which raised some concerns regarding the selection of the reported result. A detailed description of the assessment can be found in supplementary information (Supplementary Fig. S1).

## Results of the Meta-Analyses

### Primary Outcomes

#### Effects of VR Breathing Interventions on Mental Health Outcomes

There was no significant effect of VR breathing interventions on *overall* mental health outcomes (*SMD* = 0.07, *SE =* 0.08, *p =* 0.39, 95% CI [-0.10, 0.24]), indicating that participants who received a VR breathing intervention did not report significant better mental health than those who received a non-VR breathing intervention. Most studies showed small and non-significant heterogeneity, indicated by overlapping CIs and statistics (*τ*^*2*^_Level 3_ = 0.00 and *τ*^*2*^_Level 2_ = 0.00, *Q*(18) = 20.79, *p* = 0.29). *I*^2^_Level 3_ = 10.32% of the total variation is attributed to between-variance and *I*^2^_Level 2_ = 2.89% to within-variance. After removal of two influential cases (Weerdmeester ES ID 2, Rockstroh ES ID 11), the model did not change (*SMD* = 0.06, *SE =* 0.08, *p =* 0.48, 95% CI [-0.11, 0.22]).

**Fig. 3:**
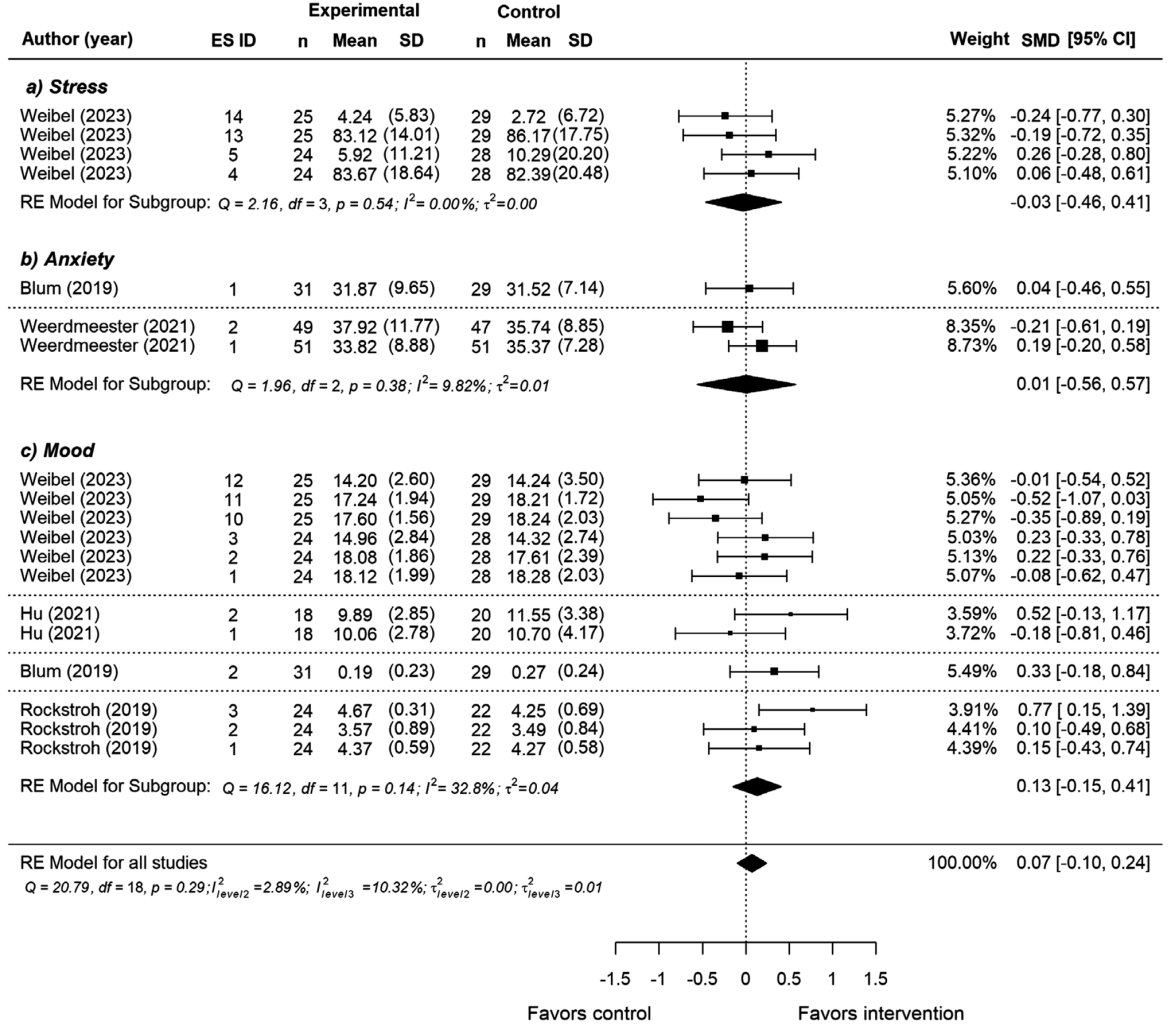
Effect of VR breathing interventions on mental health outcomes. *Note*: The forest plot shows the effect of VR-breathing interventions on *overall* mental health outcomes and separate effects for each individual outcome: (**a**) Stress, (**b**) Anxiety, and (**c**) Mood. Positive effect sizes favor the intervention, whereas negative effect sizes favor the control.

As a result of separate analyses for each outcome, we found a non-significant effect of VR on mood (*SMD* = 0.13, *SE* = 0.12, *p =* 0.33, 95% CI [-0.15, 0.41], GRADE: moderate certainty), and no significant effect on stress (*SMD* = -0.03, *SE* = 0.14, *p =* 0.86, 95% CI [-0.46, 0.41], GRADE: moderate certainty), or anxiety (*SMD* = 0.01, *SE* = 0.13, *p =* 0.96, 95% CI [-0.56, 0.57], GRADE: moderate certainty) (see Fig. 3).

#### Effects of VR HRV-Biofeedback on Mental Health Outcomes

**Fig. 4:**
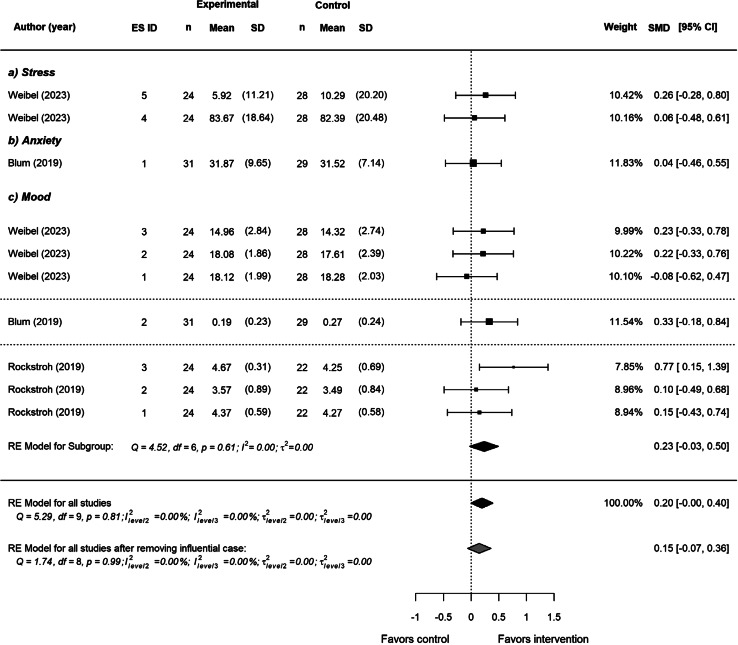
Effect of VR HRV-biofeedback on mental health outcomes. *Note*: The forest plot shows the effect of VR HRV-biofeedback on *overall* mental health outcomes, displaying separate pooled effects for mood, and the effect on *overall* mental health after removing an influential case. Positive effect sizes favor the intervention, whereas negative effect sizes favor the control.

There was no significant effect of VR HRV-Biofeedback on *overall* mental health outcomes (*SMD* = 0.20, *SE =* 0.09, *p =* 0.05, 95% CI [-0.00, 0.40]). Participants who received HRV-biofeedback via VR did not report better mental health than those using a non-VR 2D screen. Overlapping CIs and statistics indicate no significant heterogeneity in *overall* mental health outcomes (*τ*^*2*^_Level 3_ = 0.00 and *τ*^*2*^_Level 2_ = 0.00, *Q*(9) = 5.29, *p* = 0.81, *I*^2^_Level 3_ = 0.00%, *I*^2^_Level 2_ = 0.00%). After removal of an influential effect size, (Rockstroh et al., 2019, ES ID 3), we observed a reduced non-significant effect on *overall* mental health outcomes (*SMD* = 0.15, *SE =* 0.09, *p =* 0.15, 95% CI [-0.07, 0.36]). Separate analyses for mood indicated that participants receiving VR HRV-biofeedback did not report better mood than those receiving non-VR (*SMD* = 0.23, *SE =* 0.11, *p =* 0.07, 95% CI [-0.03, 0.50]). The effects on mood were slightly reduced after removal of the same influential case (*SMD* = 0.10, *SE =* 0.11, *p =* 0.38, 95% CI [-0.16, 0.36]) (see Fig. 4).

### Secondary Outcomes

#### Effects of VR Breathing Interventions on Physiological Measures of Stress

**Fig. 5:**
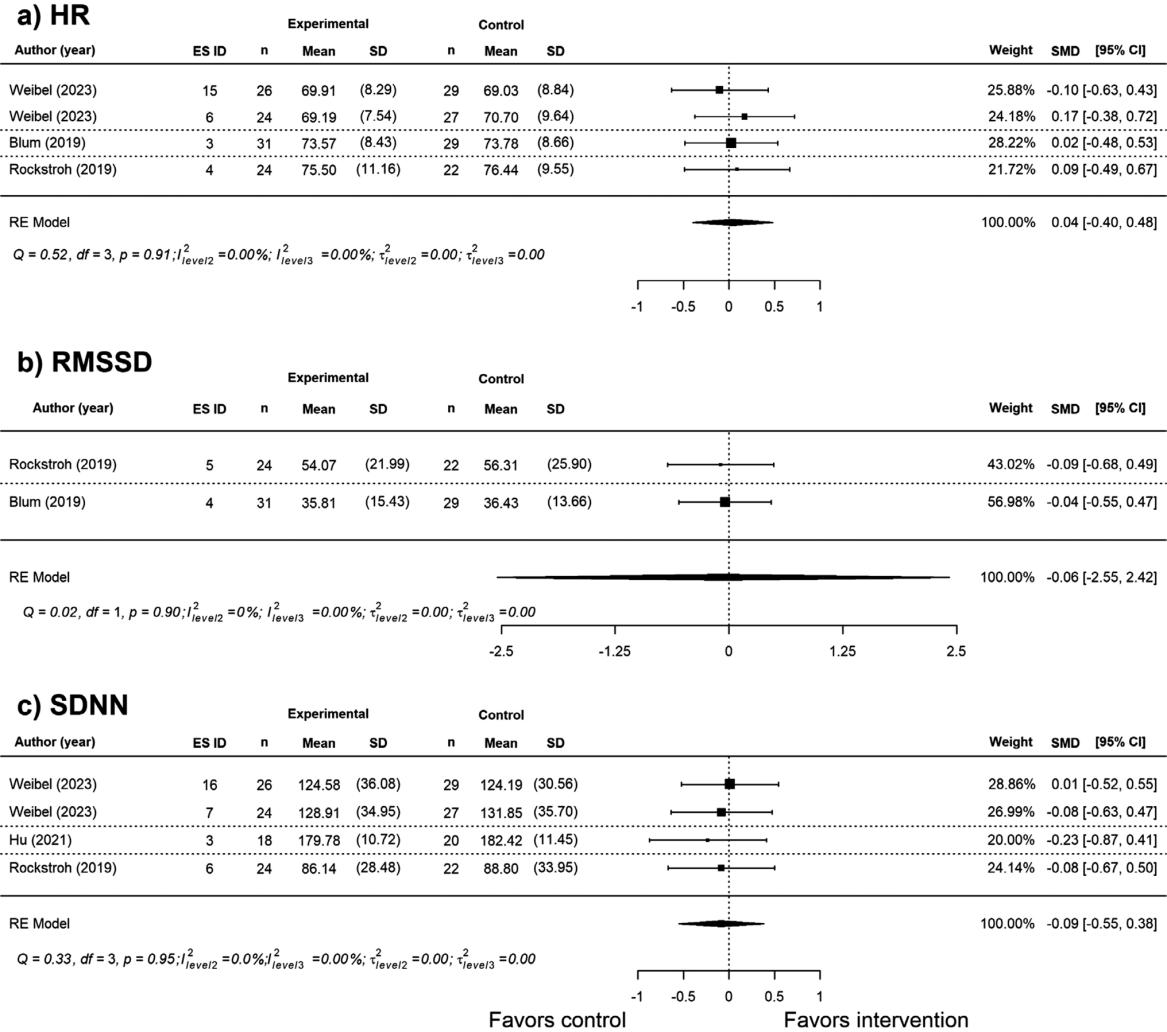
Effect of VR breathing interventions on physiological measures of stress. *Note*: The forest plot shows the effect of VR-breathing interventions on (**a**) HR, (**b**) RMSSD, and (**c**) SDNN compared with non-VR breathing intervention. Positive effect sizes favor the intervention, whereas negative effect sizes favor the control.

We examined the effects of VR breathing interventions on heart rate (HR) and two measures of heart rate variability: RMSSD (Root Mean Square of Successive Differences) and SDNN (Standard Deviation of NN intervals). A non-significant effect of VR breathing intervention was found for HR (*SMD* = 0.04, *SE =* 0.14, *p =* 0.78, 95% CI [-0.40, 0.48]), RMSSD (*SMD* = -0.06, *SE =* 0.20, *p =* 0.80, 95% CI [-2.55, 2.42], GRADE: very low certainty), and SDNN (*SMD* = -0.09, *SE =* 0.15, *p =* 0.60, 95% CI [-0.55, 0.38], GRADE: low certainty). This suggests that physiological measures of stress did not differ between VR and non-VR breathing interventions. There was no significant heterogeneity for each physiological outcome as observed by overlapping CIs and statistics (see Fig. 5).

#### Effects of VR Breathing Interventions on Liking and Future Use Outcomes

**Fig. 6:**
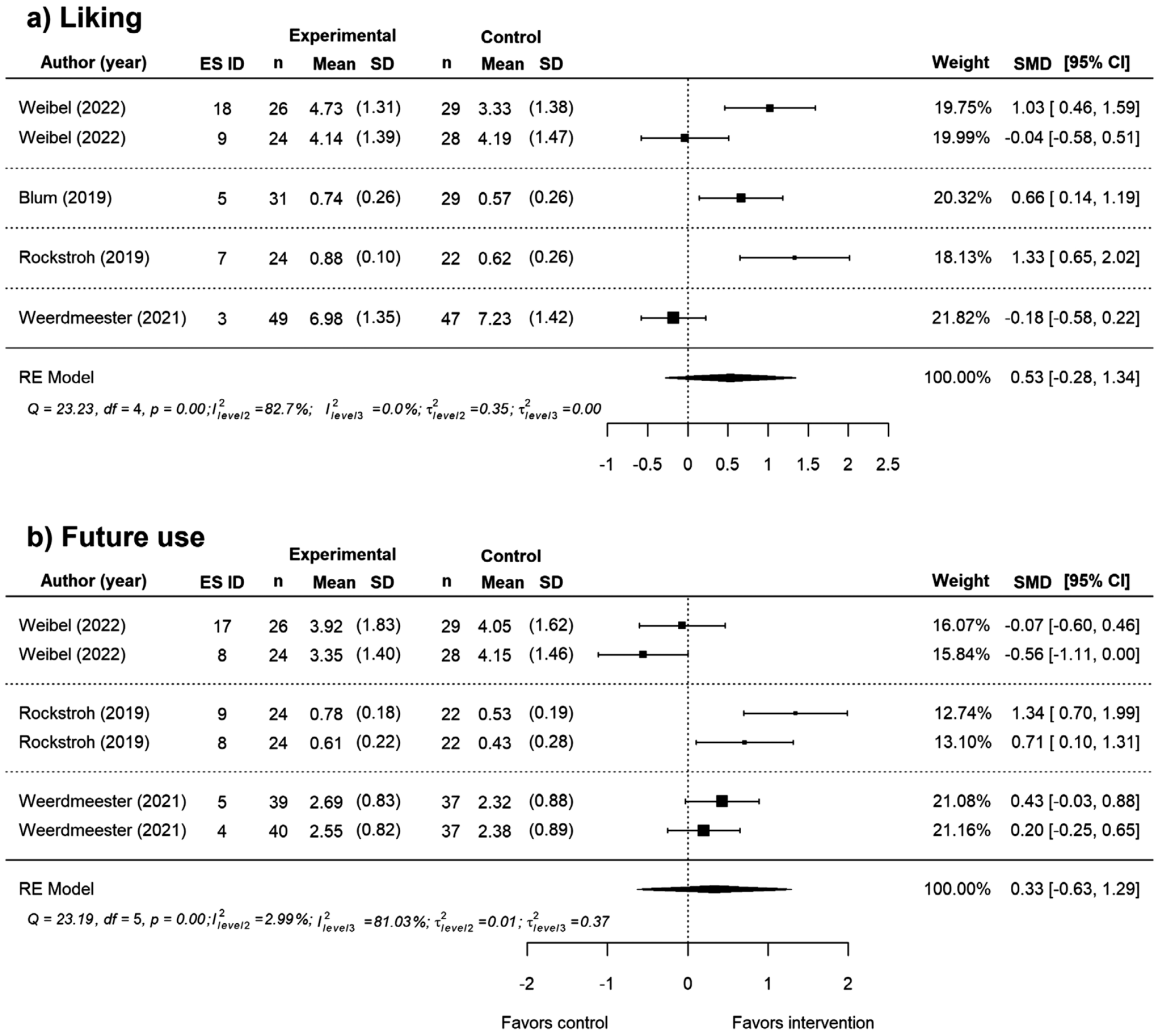
Overall Effect of VR breathing interventions on liking and future use. *Note*: The forest plot shows the overall effect of VR-breathing interventions on (**a**) Liking and (**b**) Future use compared with active control. Positive effect sizes favor the intervention, whereas negative effect sizes favor the control.

Within the selected studies, a small to moderate but non-significant effect was found on liking (*SMD* = 0.53, *SE =* 0.29, *p =* 0.14, 95% CI [-0.28, 1.34]) and future use (*SMD* = 0.33, *SE =* 0.37, *p =* 0.42, 95% CI [-0.63, 1.29], GRADE: very low certainty). Participants neither liked nor would use VR breathing interventions significantly more than non-VR breathing interventions. Liking outcomes had small statistical heterogeneity (*τ*^*2*^_Level 3_ = 0.00 and *τ*^*2*^_Level 2_ = 0.35, *Q*(4) = 23.23, *p* < 0.001, *I*^*2*^_Level 3_ = 0.00%, *I*^*2*^_Level 2_ = 82.72%) but moderate overlapping CIs. Future use showed a large statistical heterogeneity (*τ*^*2*^_Level 3_ = 0.371 and *τ*^*2*^_Level2_ = 0.013, *Q*(5) = 23.19, *p* < 0.001, *I*^*2*^_Level 3_ = 81.02%, *I*^*2*^_Level 2_ = 2.99%) and largely not overlapping CIs (see Fig. 6).

We ran individual sub-group analyses to further explore the heterogeneity of the outcome ‘future use’. Sub-group analysis of type of intervention showed that participants would not use VR HRV-biofeedback significantly more in the future than traditional 2D HRV biofeedback (*SMD *= 0.25, *SE *= 0.78, *p* = 0.78, 95% CI [-3.12, 3.63]). Furthermore, VR diaphragmatic breathing with biofeedback would not be used significantly more in the future than a paced breathing app (*SMD* = 0.31, *SE* = 0.16, *p =* 0.31, 95% CI [-1.77, 2.39]).

### Publication Bias and Influential Cases

**Fig. 7:**
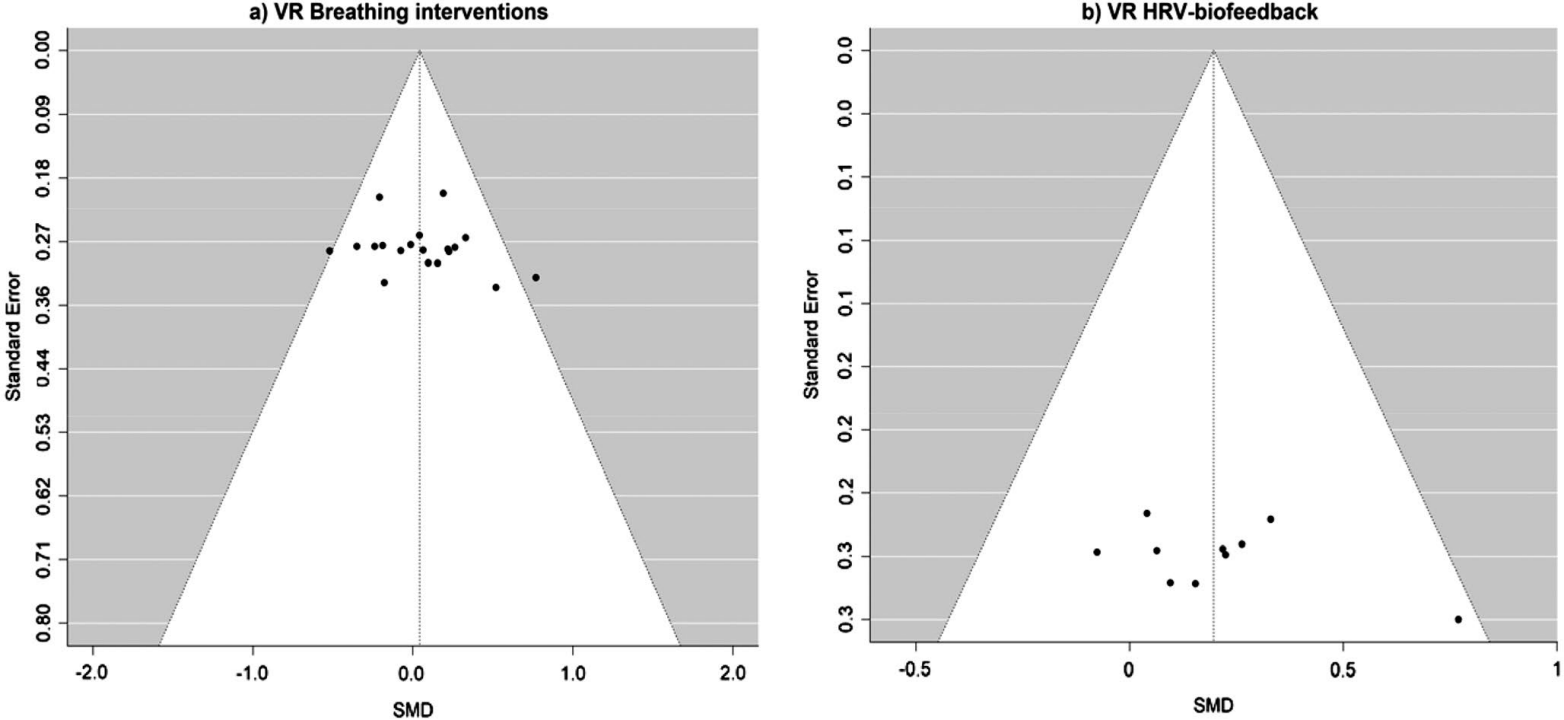
Trim-and-Fill Funnel Plot for Mental Health Outcomes. *Note*: Funnel plot with imputed missing values for (**a**) VR breathing interventions and (**b**) VR HRV-biofeedback.

Other than the influential cases on the main model and the subgroup analysis of HRV-biofeedback for mental health, no other cases were considered outliers and/or influential according to studentized residuals and cook’s distances. For *overall* mental health, trim-and-fill plot imputed estimations of effect sizes and Egger’s regression tests did not reveal publication bias (*p =* 0.41; see Fig. 7). For the VR-HRV biofeedback subgroup analysis, the funnel plot revealed an asymmetrical distribution, however, such asymmetry did not indicate publication bias, according to Eggers’s test (*p = 0.*36, see Fig. 7). There were some asymmetries in the forest plots of physiological outcomes but Eggers’s tests were not significant for HR (*p =* 0.77) and SDNN^8^ (*p =* 0.66) indicating that the asymmetry does not suggest risk for publication bias. For evaluation outcomes, the funnel plot revealed an asymmetrical distribution for liking, however, such asymmetry did not indicate publication bias, according to Eggers’s test (*p = 0.*13). The Trim-and-Fill plot imputed estimations of future use and Egger’s regression test showed no evidence of publication bias (*p =* 0.52) (see supplementary Fig. S2 and S3).

## Grade

According to GRADE, the evidence for mental health outcomes has moderate certainty primarily due to moderate imprecision. Future use and HRV outcomes were of low to very low quality due to (high) inconsistency, imprecision, and indirectness caused by the use of surrogate outcomes in HRV (See supplementary information Table S8).

## Discussion

This study investigated the effects of VR breathing interventions compared to non-VR breathing interventions on *overall* and individual mental health outcomes such as stress, mood and anxiety, as well as physiological measures of stress including HR and HRV in adults. Additionally, we analyzed liking and future use as outcomes in the selected studies. This meta-analysis found no evidence that VR breathing interventions are more effective than non-VR in improving *overall* mental health or individual mental health outcomes, with moderate quality of the evidence. Moreover, we found that sub-group analysis revealed no significant differences between VR and non-VR HRV biofeedback in *overall* mental health and mood. Furthermore, we found no significant differences between VR and non-VR breathing interventions in HR and HRV. HRV evidence was evaluated as very low to low quality. Despite small to moderate effects of VR breathing interventions on liking and future use, these differences were not statistically significant for the selected studies. Future use evidence was graded as very low quality. Overall, these results suggest that VR breathing interventions did not show significant differences compared with non-VR in mental health, physiological stress and evaluation outcomes.

These findings are consistent with prior research showing that VR-based interventions and active controls are similarly effective at improving mental health and physiological outcomes. For instance, studies comparing VR and non-VR-based mental health interventions, including evidence-based therapies, relaxation techniques, and biofeedback (meditation, relaxation techniques, and breathing interventions), have shown similar effects on anxiety at post-treatment (Dellazizzo et al., 2020; Fodor et al., 2018; Kothgassner et al., 2022; Wu et al., 2021), and on HR and HRV (Kothgassner et al., 2022). However, due to a limited number of studies that evaluated inactive control groups, the effects of VR interventions in comparison to inactive controls could not be quantified. One study found that VR-HRV biofeedback significantly improved calm mood and SDNN (Rockstroh et al., 2019). However, no significant differences were found in HR, RMSSD, positive and tired mood when compared with no treatment (Rockstroh et al., 2019). Nonetheless, these findings need to be interpreted with caution as the quality of the evidence for mental health and HRV outcomes ranges from very low to moderate. Reasons for (very) low to moderate quality include small sample sizes, large heterogeneity, and use of surrogate outcomes of mental health when it comes to HRV outcomes. Additionally, all studies showed some methodological limitations that raised concerns of bias. Among these limitations were insufficient information regarding the randomization and allocation procedures, a lack of blinding of experimenters and participants, a high level of missing data, and a lack of predefined analyses. There is a need for further high-quality research to provide robust evidence regarding the superiority of VR breathing interventions over active and inactive controls and to identify specific outcomes and moderators for which VR may have the greatest impact.

In the studies included in the meta-analysis that investigated differences in affective and physiological measures of mental health between VR and non-VR breathing interventions, no significant differences in liking and future use were observed. This contrasts with previous research indicating that VR-based mental health interventions generally increase user satisfaction and experience in pre-post designs (Kothgassner et al., 2022) and when compared to non-VR stress management and relaxation interventions (Lüddecke & Felnhofer, 2022; Velana et al., 2022). In fact, we found no evidence that participants prefer VR over non-VR interventions, nor evidence for an increased likelihood of using VR interventions in the future. Additionally, the cross-over RCT study included in our systematic review showed that VR mindful breathing was perceived as more enjoyable and satisfying than non-VR, but also showed more distractibility and fatigue (Waller et al., 2021). However, these results are not conclusive since participants were exposed to both VR and non-VR mindful breathing without a washout period. The quality of evidence on future use outcomes was very low due to significant heterogeneity and imprecise estimates. It is possible that heterogeneity in future use outcomes, particularly those from Rockstroh et al., 2021 and Weibel et al., 2023, may be associated with the specific design features of the VR breathing interventions. These components could be: (1) the type of feedback (positive, negative, continuous) (Patibanda et al., 2017); (2) the level of engagement or complexity in the feedback (Blum et al., 2019) ; (3) the level of challenge, difficulty, rewards and guidance (Weerdmeester et al., 2020); (4) the visual aesthetics of the virtual environment (Weber et al., 2021). Overall, it is plausible that the complexity of VR breathing interventions may lead to a diminished preference when compared to non-VR. Future studies could conduct a full meta-analysis of evaluation outcomes, address these methodological limitations, and explore potential factors that may influence evaluation outcomes, such as the type of breathing intervention, design features, the population characteristics, and the outcomes measured.

The lack of larger effects of VR breathing interventions over non-VR may potentially be explained by unexamined long-term effects or dose-response relationships. Prior research has suggested that a single session of non-VR breath control may be sufficient to enhance mental health and physiological indicators of stress  (Lehrer et al., 2020; Magnon et al., 2021). This may explain why both VR and non-VR breath control improve mood (Weibel et al., 2023), decrease anxiety (Blum et al., 2019; Weerdmeester et al., 2021), and reduce stress (Weibel et al., 2023), as well as increase HRV (Blum et al., 2019; Rockstroh et al., 2019; Weibel et al., 2023). However, despite the short-term benefits, VR breathing interventions may have greater effects in the long-term. Preliminary evidence has found that multi-session VR-based biofeedback interventions, which include relaxation techniques and breathing exercises, are more effective at improving psychological and physiological stress than non-VR biofeedback (Lüddecke & Felnhofer, 2022). Taking into consideration the hypothesized advantages of VR breathing interventions over non-VR, more sessions may be required to overcome the initial novelty and complexity of VR and to establish its advantages, including reduced difficulty, and increased engagement and motivation to practice breathing exercises (Lüddecke & Felnhofer, 2022). Further controlled studies are needed to evaluate the potential benefits and limitations of VR breathing interventions compared to non-VR, to identify the optimal duration and frequency for different mental health outcomes, and to investigate the mechanisms through which VR breathing exercises may offer advantages over non-VR.

Furthermore, it is plausible that VR breathing interventions may not have a significant advantage over non-VR. VR may not provide the theorized benefits, such as increased motivation, engagement, and a reduction in distractions and difficulty during breathing interventions. Nevertheless, these advantages may be reflected *during* the intervention process, thus moderating the treatment effects of VR breathing interventions. Previous research has shown that greater involvement in a virtual environment exposure treatment is linked to improved treatment response for public speaking fear (Price et al., 2011). In some of the studies included in our meta-analysis, VR breathing interventions, compared to non-VR, resulted in higher focused attention (Rockstroh et al., 2019), higher immersive adaptation (Weibel et al., 2023), and less distraction (Rockstroh et al., 2019). However, only one study examined the relationship between engagement and treatment response, and found that greater engagement was associated with greater decrease in anxiety, but only for a non-VR paced breathing intervention (Weerdmeester et al., 2021). It is recommended that further studies investigate the role of in-session immersion, presence, attention, distraction, engagement, and perceived difficulty of breathing exercises on treatment response to VR breathing interventions. Gaining a better understanding of whether VR implementations of breathing interventions may have advantages, and if so, which and how, is essential in light of the potentially higher sustainability of breathing interventions, especially among those who benefit from them, to promote mental health, as well as to prevent the onset or worsening of mental health concerns in diverse populations.

This systematic review has notable strengths. It is the first study to evaluate the effects of VR breathing interventions on mental health, physiological measures of stress, and user experience outcomes using RCTs as the gold standard of evidence. Additionally, the review employs a multi-level approach, allowing for accurate consideration of multiple effect sizes within the same study and ensuring precise estimates. However, it also has several limitations. First, the small number of studies included in the analysis may have resulted in a lack of power to detect effects. In the field of mental health research, VR is an emerging technology. However, the combination of VR with breathing interventions is relatively new. We expect an increase of research in the field in which more randomized controlled studies with robust methodologies are needed. Second, we combined VR breath awareness and breath control interventions, which may result in different short-term and long-term effects. Breath control interventions directly increasing vagal activity (Lehrer et al., 2020) may have a faster effect on mental health outcomes and physiological stress (Balban et al., 2023), while breath control and breath awareness interventions aimed at improving adaptive functioning (each through different mechanisms) may result in larger long-term effects (Hayes et al., 2012). Third, the inclusion of studies employing a variety of breathing interventions (e.g., diaphragmatic breathing versus paced breathing) as well as different modalities (VR versus non-VR) makes it difficult to draw conclusive conclusions regarding the added value of VR. As the number of VR breathing studies increases, it is recommended to examine the effects of the same type of breathing intervention delivered in different modalities in order to better understand VR effects.

## Conclusions

In spite of the limitations of our study, its findings importantly contribute to the literature on the use of VR in breathing interventions, emphasizing the importance of further high-quality research in this area. Future studies should aim to address methodological limitations, investigate potential factors that can enhance the effectiveness of VR in comparison with active and inactive controls, and evaluate the long-term effects of multi-session interventions. In conclusion, the results of our study suggest that there is no evidence that VR-based breathing interventions are more effective than non-VR interventions in enhancing mental health outcomes, HR, and HRV. Additionally, the level of certainty regarding mental health outcomes is considered moderate. There is, however, a need for further research in order to fully understand the potential of VR in this field, and to determine its suitability for specific populations.

### Electronic Supplementary Material

Below is the link to the electronic supplementary material.

Supplementary Material 1

## Data Availability

Data are available upon request. R code is available at: https://osf.io/tvr5m/.

## Footnotes

1 Free text terms allow for a more flexible and unstandardized search based on exact words or phrases used in articles.

2 Indexed terms are standardized terms used by databases to categorize and tag articles.

3 According to our sensitivity analysis, none of our models differed in overall effect when including inactive control group effect sizes (see supplementary information). In addition, inactive control group effect sizes were derived from a study for which an active control group effect size was included.

4 According to our sensitivity analysis, none of our models differed in overall effects when including effect sizes combining mindful breathing and diaphragmatic breathing after day 7 (not reported).

5 We choose RMSSD and SDNN as they are two of the most common measures of overall HRV and are less affected by respiratory influences and population characteristics than frequency-domain measures (Laborde et al., 2017). In addition, frequency-domain measures were not comparable across studies, and thus not suited for analysis, as some studies investigated breathing rates in the low frequency range, while spontaneous breathing occurs in the high frequency range.

6 Authors were contacted for the request of data from the first time period; however, no response was received.

7 We excluded the cross-over trial from the risk of bias assessment because our primary focus was on evaluating the first period of the trial. This was due to our expectation of carryover effects and exposure to both VR and non-VR conditions.

8 The funnel plot for RMSSD was not created due to the small number of effect sizes.
