# Supplementary material for: Virtual Reality Breathing Interventions for Mental Health: A Systematic Review and Meta-Analysis of Randomized Controlled Trials
Source: Appl Psychophysiol Biofeedback. 2024 Jan 18;49(1):1–21. doi: 10.1007/s10484-023-09611-4 (PMC10869395; doi:10.1007/s10484-023-09611-4)
Supplement: Supplementary file 1 — Supplementary Material 1 [file 10484_2023_9611_MOESM1_ESM.docx]

# **Supplementary information**

## **Supplementary Table S1**

Sensitivity Analyses of the Effects of VR Breathing Interventions on Primary and Secondary Outcomes

|  |  |  | | Effect size | | | Heterogeneity | | | | |
| --- | --- | --- | --- | --- | --- | --- | --- | --- | --- | --- | --- |
|  |  | **N Cluster** | **n ES** | **SMD** | **95% CI** | ***p*** | ***I* ^2^**  **_Level 3_** | ***I* ^2^**  **_Level 2_** | ***τ^2^***  **_Level 3_** | ***τ^2^***  **_Level 2_** | ***Q ( p)*** |
| PRIMARY OUTCOMES | | | | | | | | | | |  |
| a) Including only active control | *Overall* mental health | 5 | 19 | 0.07 | -0.10 0.24 | 0.39 | 10.32% | 2.89 % | 0.01 | 0.00 | 20.79  (0.29) |
|  | Stress | 1 | 4 | -0.03 | -0.46 0.41 | 0.86 |  | 0% |  | 0.00 | 2.16  (0.54) |
|  | Anxiety | 2 | 3 | 0.01 | -0.56 0.57 | 0.96 | 0% | 09.82% | 0.00 | 0.01 | 1.96  (0.38) |
|  | Mood | 4 | 12 | 0.13 | -0.15 0.41 | 0.33 | 18.7% | 14.1% | 0.02 | 0.02 | 16.12  (0.14) |
| b) Including inactive control | *Overall* mental health | 5 | 22 | 0.13 | -0.09 0.35 | 0.23 | 24.61% | 12.28 | 0.03 | 0.01 | 34.24  (0.03) |
|  | Stress | 1 | 4 | -0.03 | -0.46 0.41 | 0.86 |  | 0% |  | 0.00 | 2.16  (0.54) |
|  | Anxiety | 2 | 3 | 0.01 | -0.56 0.57 | 0.96 | 0% | 9.82% | 0.00 | 0.01 | 1.96  (0.38) |
|  | Mood | 4 | 15 | 0.19 | -0.13 0.51 | 0.21 | 26% | 24.1% | 0.04 | 0.04 | 28.10  (0.01) |
| c) Removing Weerdmeester et al., 2021 | *Overall* mental health | 4 | 17 | 0.11 | -0.11 0.33 | 0.31 | 18.45% | 0% | 0.02 | 0.00 | 18.73  (0.28) |
|  | Stress | 1 | 4 | -0.03 | -0.46 0.41 | 0.86 |  | 0% |  | 0.00 | 2.16  (0.54) |
|  | Mood | 4 | 12 | 0.13 | -0.15 0.41 | 0.33 | 18.7% | 14.1% | 0.02 | 0.02 | 16.12  (0.14) |
| SECONDARY OUTCOMES | | | | | | | | | | | |
| a) Including only active control | HR | 3 | 4 | 0.04 | -0.40 0.48 | 0.78 | 0% | 0% | 0.00 | 0.00 | 0.52  (0.91) |
|  | RMSSD | 2 | 2 | -0.06 | -2.55 2.42 | 0.80 | 0% | 0% | 0.00 | 0.00 | 0.02  (0.90) |
|  | SDNN | 3 | 4 | -0.09 | -0.55 0.38 | 0.60 | 0% | 0% | 0.00 | 0.00 | 0.33  (.95) |
|  | Liking | 4 | 5 | 0.53 | -0.28 1.34 | 0.14 | 0% | 82.72% | 0.00 | 0.35 | 23.23  (< .01) |
|  | Future use | 3 | 6 | 0.33 | -0.63 1.29 | 0.42 | 81.1% | 2.99% | 0.37 | 0.01 | 23.19  (< .01) |
| b) Including inactive control | HR | 3 | 5 | 0.07 | -0.28 0.42 | 0.60 | 0% | 0% | 0.00 | 0.00 | 0.80  (0.94) |
|  | RMSSD | 2 | 3 | 0.06 | -0.64 0.77 | 0.74 |  |  | 0.00 | 0.00 | 1.41  (0.49) |
|  | SDNN | 3 | 5 | 0.20 | -0.63 1.02 | 0.54 | 0% | 79.53 | 0.00 | 0.35 | 17.76  (< .01) |
|  | Liking | 4 | 6 | 0.69 | -0.28 1.66 | 0.13 | 47.70% | 40.35 | 0.32 | 0.27 | 37.74  (< .01) |
| c) Removing Weerdmeester et al., 2021 | Liking | 3 | 4 | 0.73 | -0.20 1.6 | 0.09 | 0% | 74.54% | 0.00 | 0.25 | 11.52  (< .01) |
|  | Future use | 2 | 4 | 0.35 | -1.75 2.45 | 0.63 | 83.93 | 6.73 | 0.79 | 0.06 | 22.44  (< .01) |

Note. Table showing the effect of VR breathing interventions on primary and secondary outcomes in comparison with a) only active control groups, b) including inactive control groups, and c) after removing Weerdmeester et al., 2021. We removed Weerdmeester et al., 2021 to minimize confounding variables and isolate the effects of VR. In this study, both VR and non-VR delivery modes were used to compare two different types of breathing interventions (e.g., diaphragmatic vs. paced breathing.

## **Supplementary Table S2**

Search Strategy in PubMed

| **Search** | **Query** |
| --- | --- |
| **#3** | **#1 AND #2 Most Recent** |
| **#2** | **"Biofeedback, Psychology"[Mesh] OR "Breathing Exercises"[Mesh] OR "Respiration"[Mesh] OR "Respiratory Sinus Arrhythmia"[Mesh] OR "breath*"[tiab] OR "respirat*"[tiab] OR "pranayama"[tiab] OR "diaphragm training"[tiab] OR "shamata"[tiab] OR "biofeedback"[tiab] OR "bio feedback"[tiab] OR "buteyko"[tiab] OR "papworth"[tiab] OR "diaphragm*"[tiab] Most Recent** |
| **#1** | **"Virtual Reality"[Mesh] OR "Virtual Reality Exposure Therapy"[Mesh] OR "Augmented Reality"[Mesh] OR "Virtual Realit*"[tiab] OR "augmented realit*"[tiab] OR "Mixed Realit*"[tiab] OR "VR"[tiab] OR "virtual environment*"[tiab] Most Recent** |

## **Supplementary Table S3**

Search Strategy in PsycINFO

| **Search** | **Query** |
| --- | --- |
| #3 | **#1 AND #2** |
| #2 | DE "Biofeedback" OR DE "Biofeedback Training" OR DE "Respiration" OR TI(“breath*” OR “respirat*” OR “pranayama” OR "diaphragm training” OR “shamata” OR “biofeedback” OR “bio feedback” OR “buteyko” OR “papworth” OR “diaphragm*”) OR AB(“breath*” OR “respirat*” OR “pranayama” OR "diaphragm training” OR “shamata” OR “biofeedback” OR “bio feedback” OR “buteyko” OR “papworth” OR “diaphragm*”) OR KW(“breath*” OR “respirat*” OR “pranayama” OR "diaphragm training” OR “shamata” OR “biofeedback” OR “bio feedback” OR “buteyko” OR “papworth” OR “diaphragm*”) ) |
| #1 | DE "Virtual Reality" OR DE "Augmented Reality" OR DE "Virtual Reality Exposure Therapy" OR TI(“Virtual Realit*” OR “augmented realit*” OR “Mixed Realit*” OR “VR” OR “virtual environment*”) OR AB(“Virtual Realit*” OR “augmented realit*” OR “Mixed Realit*” OR “VR” OR “virtual environment*”) OR KW(“Virtual Realit*” OR “augmented realit*” OR “Mixed Realit*” OR “VR” OR “virtual environment*”) |

## **Supplementary Table S4**

Search Strategy in Web of Science

| **Search** | **Query** |
| --- | --- |
| #3 | **#1 AND #2** |
| #2 | TS=(“breath*” OR “respirat*” OR “pranayama” OR "diaphragm training” OR “shamata” OR “biofeedback” OR “bio feedback” OR “buteyko” OR “papworth” “diaphragm*”) |
| #1 | TS=(“Virtual Realit*” OR “augmented realit*” OR “Mixed Realit*” OR “VR” OR “virtual environment*”) |

## **Supplementary Table S5**

Search Strategy in Cochrane Library

| **Search** | **Query** |
| --- | --- |
| #3 | **#1 AND #2** |
| #2 | ((breath*) OR (respirat*) OR (pranayama) OR (diaphragm NEXT training) OR (shamata) OR (biofeedback) OR (bio NEXT feedback) OR (buteyko) OR (papworth) (diaphragm*)):ti,ab,kw |
| #1 | ((Virtual NEXT Realit*) OR (augmented NEXT realit*) OR (Mixed NEXT Realit*) OR (VR) OR (virtual NEXT environment*)):ti,ab,kw |

## **Supplementary Table S6**

Search Strategy in Embase

| **Search** | **Query** |
| --- | --- |
| #4 | #3 NOT ('conference abstract'/it OR 'conference paper'/it) |
| #3 | **#1 AND #2** |
| #2 | 'biofeedback'/de OR 'breathing exercise'/exp OR 'breathing'/exp OR 'respiratory sinus arrhythmia'/exp OR (‘breath*’ OR ‘respirat*’ OR ‘pranayama’ OR ‘diaphragm training’ OR ‘shamata’ OR ‘biofeedback’ OR ‘bio feedback’ OR ‘buteyko’ OR ‘papworth’ OR ‘diaphragm*’):ti,ab,kw |
| #1 | 'virtual reality'/exp OR 'virtual reality exposure therapy'/exp OR 'augmented reality'/exp OR (‘Virtual Realit*’ OR ‘augmented realit*’ OR ‘Mixed Realit*’ OR ‘VR’ OR ‘virtual environment*’):ti,ab,kw |

## **Supplementary Table S7**

Search Strategy in Scopus

| **Search** | **Query** |
| --- | --- |
| #4 | ( TITLE-ABS-KEY ( "Virtual Realit*" OR "augmented realit*" OR "Mixed Realit*" OR "VR" OR "virtual environment*" ) ) AND ( TITLE-ABS-KEY ( "breath*" OR "respirat*" OR "pranayama" OR "diaphragm training" OR "shamata" OR "biofeedback" OR "bio feedback" OR "buteyko" OR "papworth" OR "diaphragm*" ) ) AND ( EXCLUDE ( DOCTYPE , "cp" ) OR EXCLUDE ( DOCTYPE , "cr" ) ) |
| #3 | **#1 AND #2** |
| #2 | TITLE-ABS-KEY (“breath*” OR “respirat*” OR “pranayama” OR “diaphragm training” OR “shamata” OR “biofeedback” OR “bio feedback” OR “buteyko” OR “papworth” OR “diaphragm*”) |
| #1 | TITLE-ABS-KEY (“Virtual Realit*” OR “augmented realit*” OR “Mixed Realit*” OR “VR” OR “virtual environment*”) |

## **Supplementary Fig. S1**

Cochrane Risk of Bias Assessment (detailed) for RCTs

**
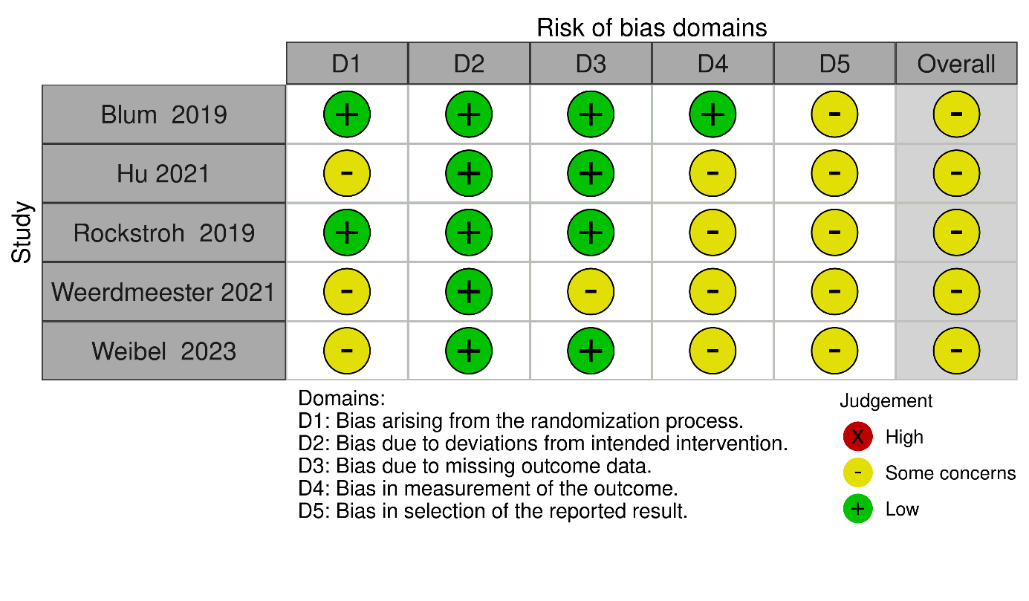
**

Note. This Fig. was created and adapted using the online Risk-of-bias Visualization (robvis) tool (McGuinness & Higgins, 2020).

## **Supplementary Fig. S2**

Funnel Plots of Physiological Measures of Stress


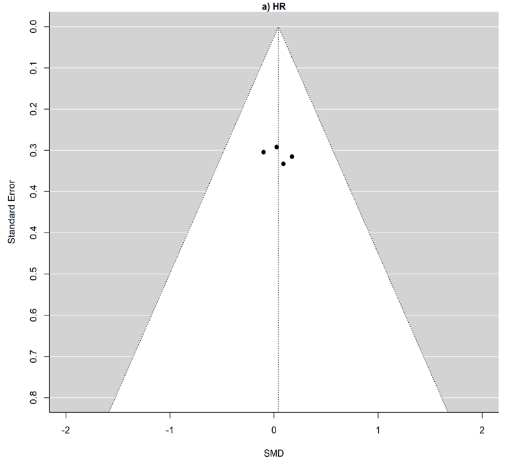

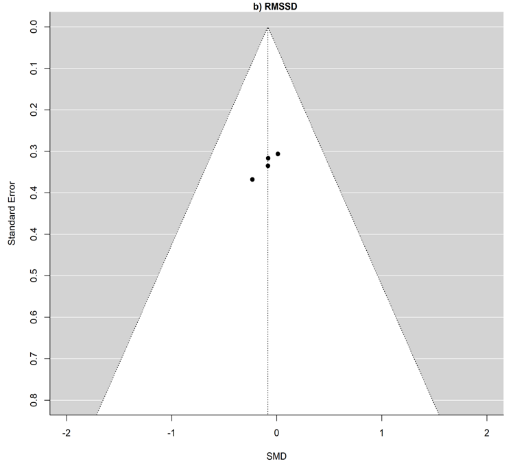


Note. Funnel plots of a) HR and b) RMSSD

## **Supplementary Fig. S3**

Funnel Plots of Liking and Future Use Outcomes


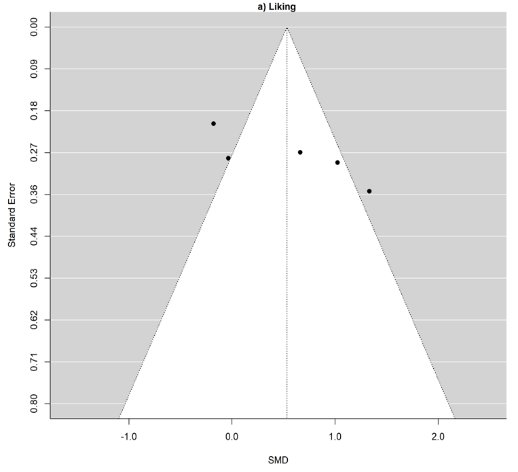

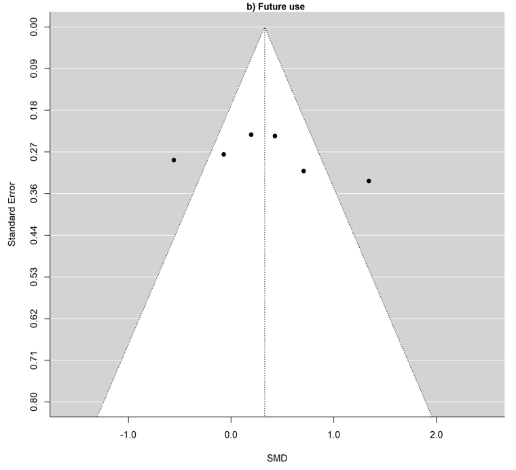


Note. Funnel plots of a) Liking and b) Future use

## **Supplementary Table S8**

GRADE Summary of Evidence

| **Summary of findings:** | | | | | | |
| --- | --- | --- | --- | --- | --- | --- |
| **Virtual reality breathing intervention compared to Non-Virtual reality breathing intervention for mental health in young adults and adults** | | | | | | |
| **Patient or population:** mental health in young adults and adults  **Setting:** varied (university lab, hospital)  **Intervention:** Virtual reality breathing intervention  **Comparison:** Non-Virtual reality breathing intervention | | | | | | |
| Outcomes | **Anticipated absolute effects^*^** (95% CI) | | Relative effect (95% CI) | № of participants (studies) | Certainty of the evidence (GRADE) | Comments |
|  | **Risk with Non-Virtual reality breathing intervention** | **Risk with Virtual reality breathing intervention** |  |  |  |  |
| Self-reported Stress assessed with: VAS follow-up: mean 1 days | - | SMD **0.03 SD lower** (0.46 lower to 0.41 higher) | - | 106 (1 RCT) | ⨁⨁⨁◯ Moderate^a,b^ |  |
| Anxiety assessed with: STAI-S Scale from: 20 to 80 follow-up: range 1 days to 2 weeks | - | SMD **0.01 SD higher** (0.56 lower to 0.57 higher) | - | 162 (2 RCTs) | ⨁⨁⨁◯ Moderate^a,b^ |  |
| Mood  assessed with: Diverse scales  Scale from: 0 to 50 follow-up: mean 1 days | - | SMD **0.13 SD higher** (0.15 lower to 0.41 higher) | - | 250 (4 RCTs) | ⨁⨁⨁◯ Moderate^a,b^ |  |
| Future use assessed with: Diverse scales  Scale from: 0 to 7 follow-up: mean 1 | - | SMD **0.33 SD higher** (0.63 lower to 1.29 higher) | - | 230 (3 RCTs) | ⨁◯◯◯ Very low^b,c,d^ |  |
| Heart rate variability (RMSSD) | - | SMD **0.06 SD lower** (2.55 lower to 2.42 higher) | - | 106 (2 RCTs) | ⨁◯◯◯ Very low^b,d,e^ |  |
| Heart rate variability (SDNN) | - | SMD **0.09 SD lower** (0.55 lower to 0.38 higher) | - | 190 (3 RCTs) | ⨁⨁◯◯ Low^a,b,e^ |  |
| Nausea | Nausea was reported in two studies via a VAS and the UTAUT questionnaire. In Weerdmeester nausea was only reported for the intervention group. Not possible to pool data. | |  | 156  (2 RCTs) | ⨁⨁⨁⨁ High^a^ |  |
| ***The risk in the intervention group** (and its 95% confidence interval) is based on the assumed risk in the comparison group and the **relative effect** of the intervention (and its 95% CI).  **CI:** confidence interval; **SMD:** standardised mean difference | | | | | | |
| **GRADE Working Group grades of evidence** **High certainty:** we are very confident that the true effect lies close to that of the estimate of the effect. **Moderate certainty:** we are moderately confident in the effect estimate: the true effect is likely to be close to the estimate of the effect, but there is a possibility that it is substantially different. **Low certainty:** our confidence in the effect estimate is limited: the true effect may be substantially different from the estimate of the effect. **Very low certainty:** we have very little confidence in the effect estimate: the true effect is likely to be substantially different from the estimate of effect. | | | | | | |

Explanations

a. Downgraded 1 for imprecision due to small sample size

b. Only downgraded for small sample size in imprecision

c. Downgraded 1 for inconsistency due to large heterogeneity

d. Downgraded 2 for imprecision due to large width of CIs and small sample size

e. Downgraded 1 for indirectness due to use of surrogate outcomes
